# A Rare Case of Non-Gestational Metastatic Ovarian Choriocarcinoma: Case Report and Literature Review With a Special Emphasis on Imaging

**DOI:** 10.7759/cureus.13121

**Published:** 2021-02-04

**Authors:** Abdul Sattar Anjum, Hamza Maqsood, Shifa Younus, Sadia Anjum, Maham Fatima

**Affiliations:** 1 Radiology, Nishtar Medical University, Multan, PAK; 2 Cardiology, Nishtar Medical University, Multan, PAK; 3 Internal Medicine, Nishtar Medical University, Multan, PAK

**Keywords:** gestational, ovarian choriocarcinoma, mdct, ct angiography

## Abstract

Non-gestational choriocarcinoma of the ovary is a very rare neoplasm. It carries a worse prognosis as compared to gestational choriocarcinoma (GCC). Here, we report a case of non-gestational ovarian choriocarcinoma. The patient initially presented in a medical emergency with abdominal pain, a feeling of heaviness in the lower abdomen, cough, and dyspnea. The patient had four healthy children, and the last childbirth was five years ago. There was no history of any abortion or stillbirth in the past four years. A highly vascular left adnexal mass was observed on ultrasound abdomen and pelvis. Compute CT chest, abdomen, and pelvis were performed, which revealed metastatic left ovarian choriocarcinoma features. It also showed vascular metastases of the carcinoma in the kidneys, liver, and lungs. We report this case specifically emphasizing ultrasound, multidetector computed tomography (MDCT), and CT angiography findings.

## Introduction

Germ cell malignancies represent 15% of ovarian cancers in Asian and African-American nations [1]. There are three ways by which ovarian choriocarcinoma can arise. These include primary tumors associated with ovarian pregnancy, a metastatic carcinoma from primary gestational choriocarcinoma in other parts of the genital tract, and a germ cell tumor admixed with other neoplastic germ cell elements [2].

Non-gestational choriocarcinoma is usually a component of mixed germ cell tumors (MGCTs). MGCTs include immature teratoma, endodermal sinus tumor, embryonal carcinoma, and dysgerminoma [3]. Isolated non-gestational choriocarcinoma of the ovary is an extremely rare neoplasm. It has a worse prognosis when compared with gestational choriocarcinoma (GCC) [4].

## Case presentation

**Figure 1:**
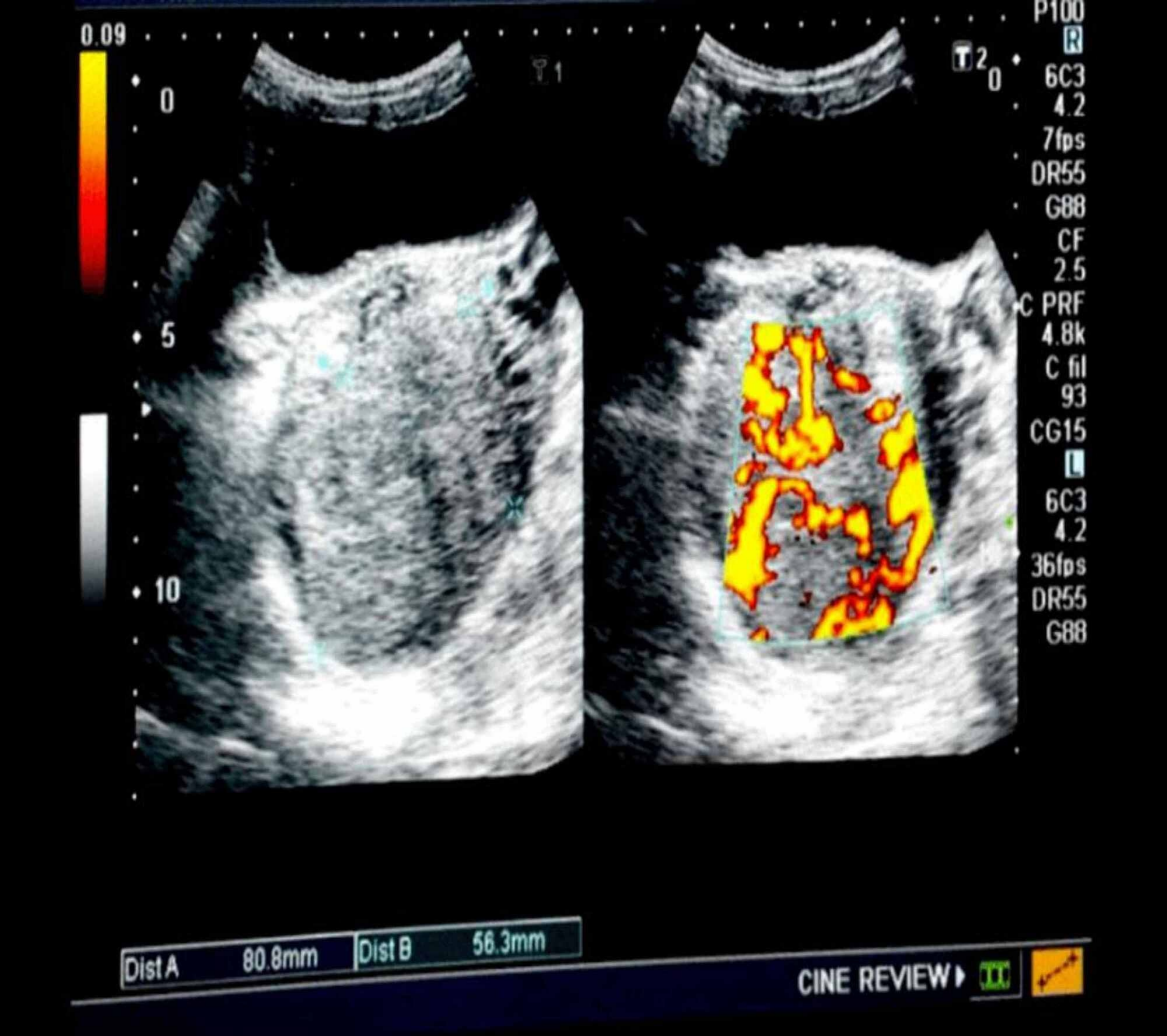
Pelvic ultrasound showing an echogenic mass in the left adnexal region. Doppler shows the mass to be highly vascular

Here, we report a case of a 36-year-old female who presented in a medical emergency with complaints of abdominal pain for six months, a feeling of heaviness in the lower abdomen for three months, cough, and shortness of breath month. The patient was para four, and the last childbirth was five years ago. There was no history of any pregnancy or miscarriage in the past four years. She had a history of 19 pints of blood transfusion in the past six months. On general physical examination, we found marked pallor and emaciation. Her abdominal palpation revealed tenderness in the left hemipelvis. As far as her labs are concerned, a complete blood count (CBC) showed hemoglobin (Hb) of 4 g/dl. Total leucocyte count (TLC), platelets, prothrombin time (PT), and activated partial thromboplastin time (aPTT) were in the normal range. Serum beta-human chorionic gonadotropin (hCG) turned out to be 6000 IU/ml. Abdomen and pelvis ultrasonography (USG) showed an echogenic mass in the left adnexal region that appeared highly vascular on Doppler ultrasound (Figure 1). The uterus was morphologically normal.

**Figure 2:**
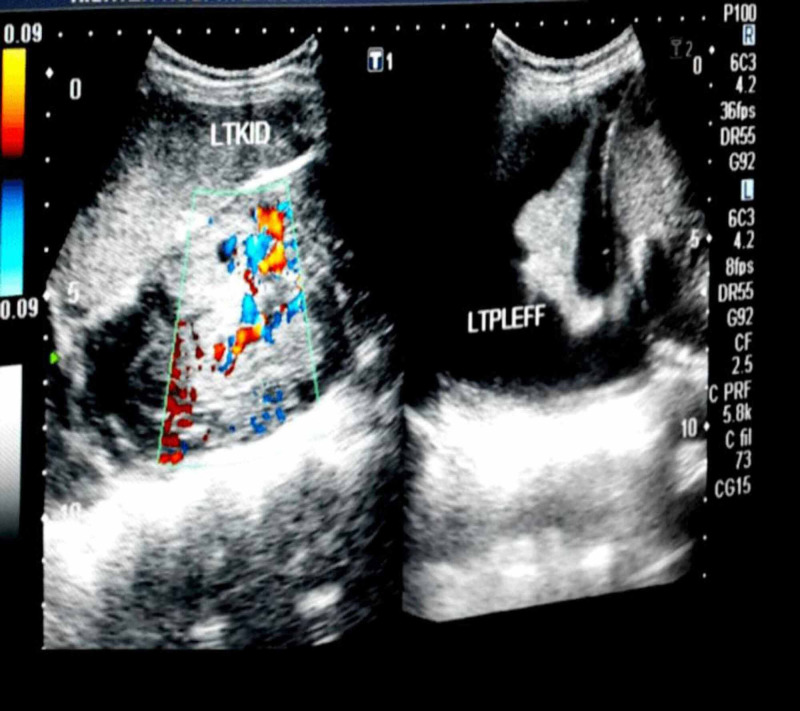
Ultrasound abdomen showing a vascular mass in the left kidney and a left pleural effusion

We found a highly vascular echogenic mass in the left kidney with a subcapsular collection. USG also revealed a left-sided pleural effusion (Figure 2).

**Figure 3:**
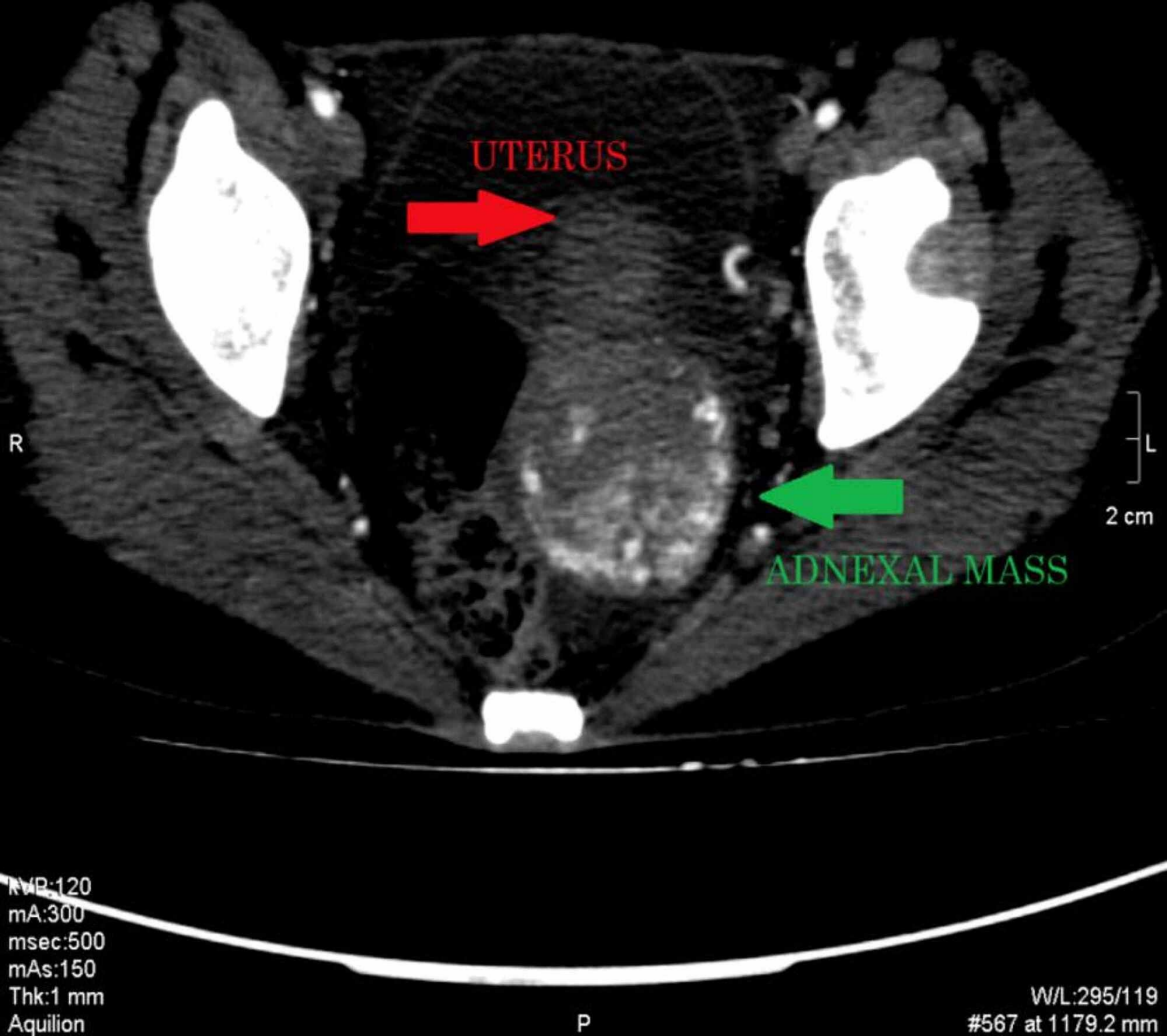
CECT pelvis axial section showing a large highly vascular mass in the left adnexal region extending up to the posterior cul-de-sac displacing the uterus anteriorly CECT: contrast-enhanced computed tomography

**Figure 4:**
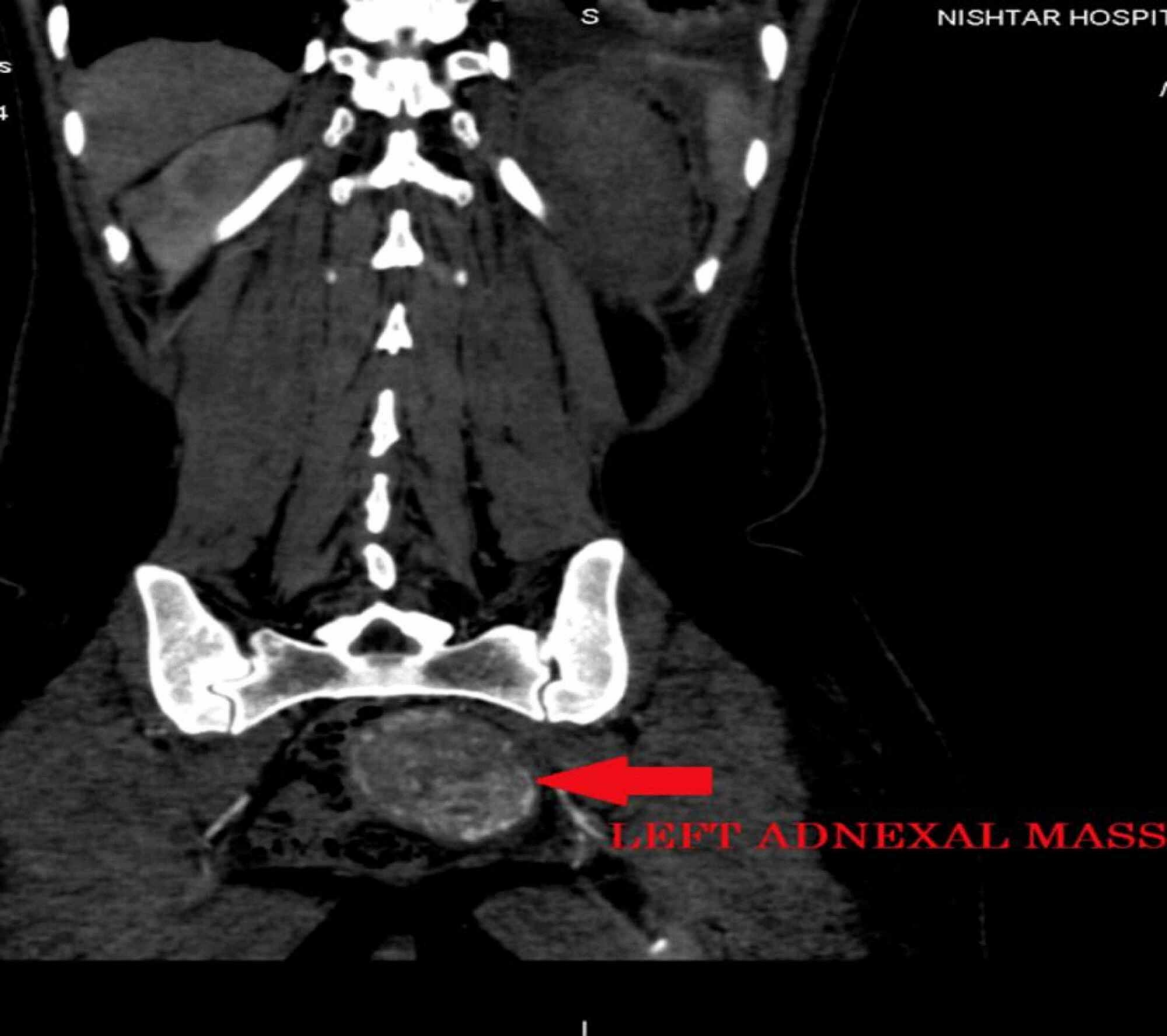
Coronal reformat CT showing an enhancing left adnexal mass CT: computed tomography

**Figure 5:**
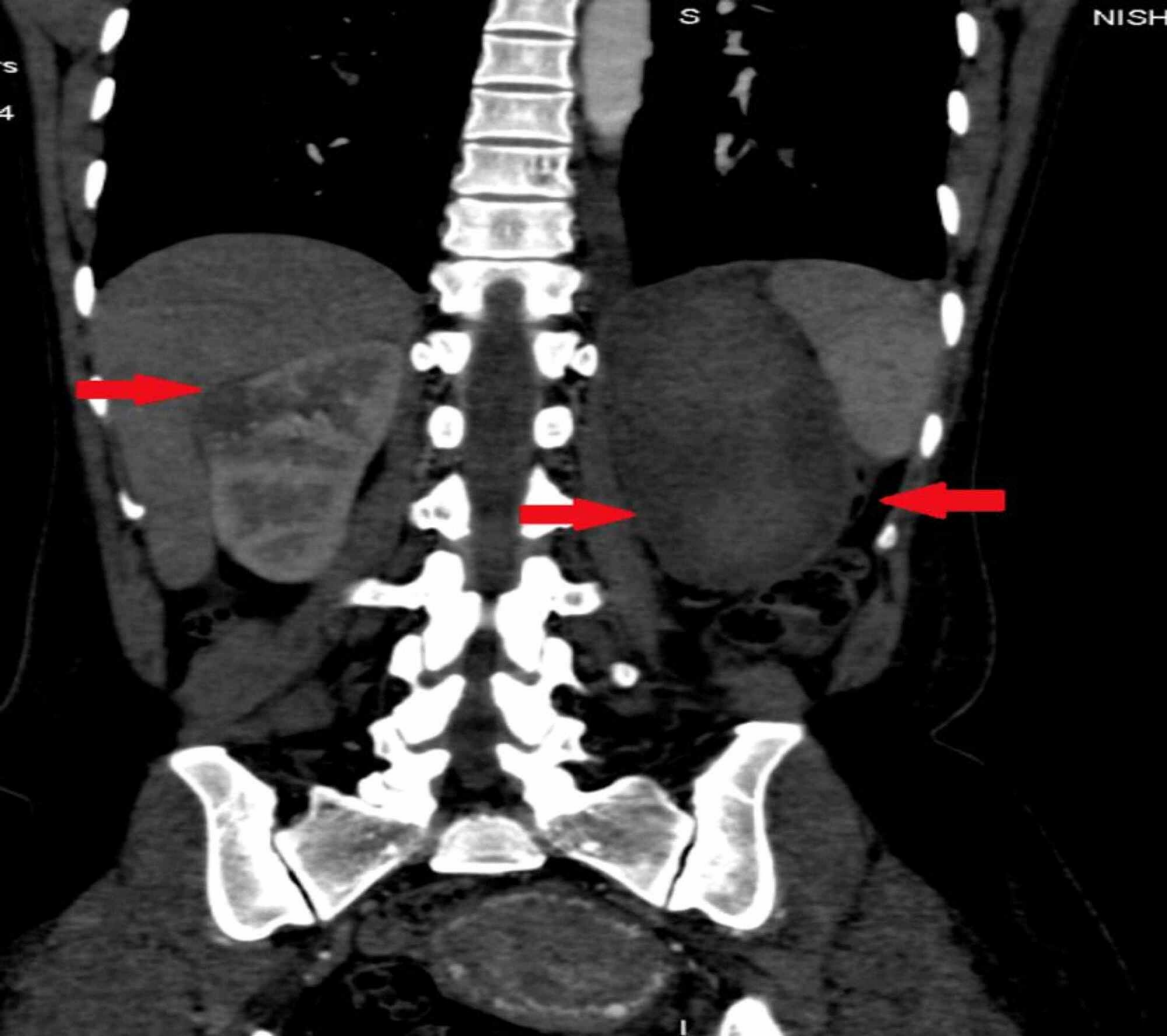
CT abdomen coronal reformat showing a soft tissue density mass in the kidney and large subcapsular collection (hematoma) in the left kidney CT: computed tomography

**Figure 6:**
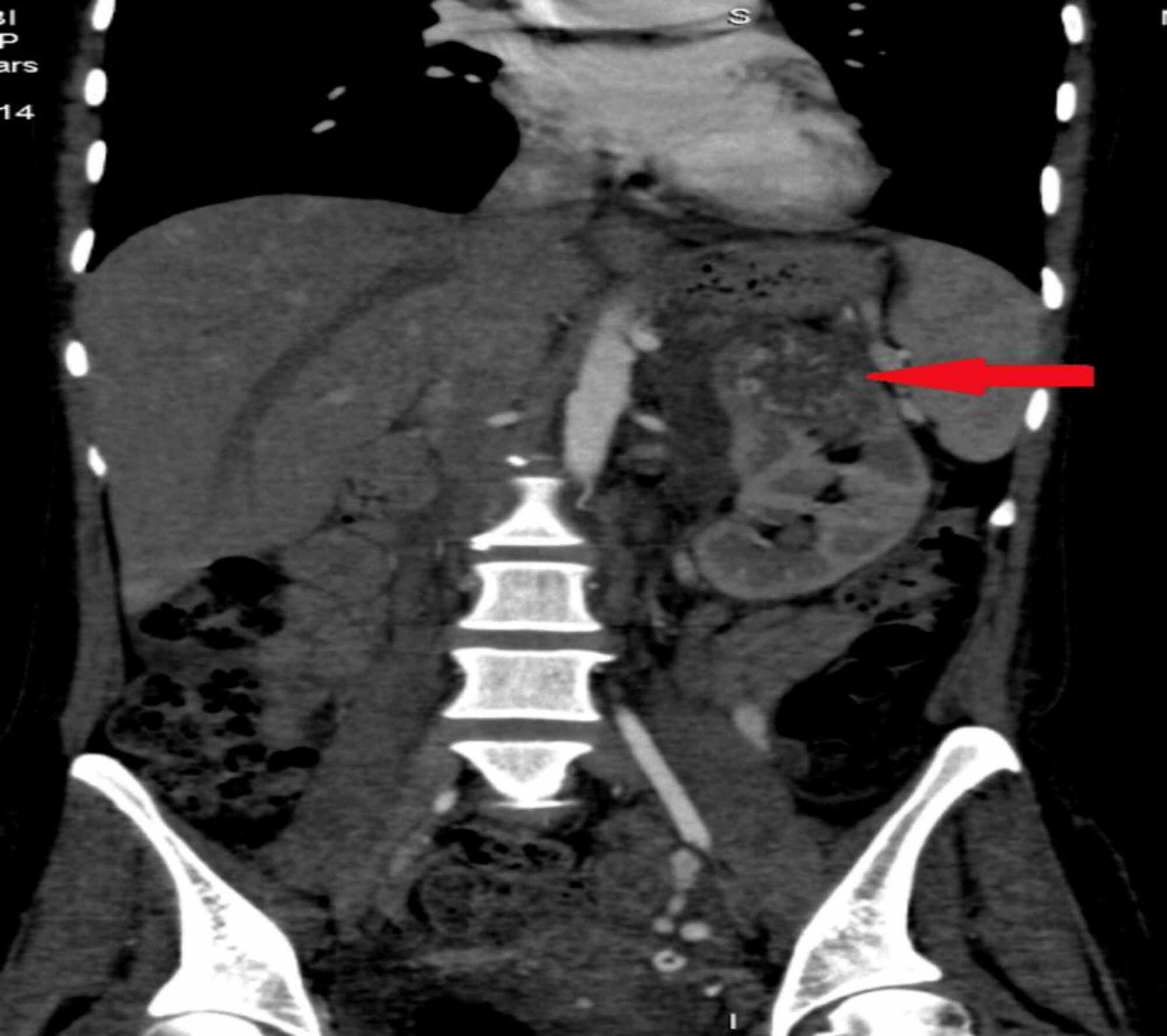
A similar enhancing soft tissue density lesion was seen on the upper pole of the left kidney

**Figure 7:**
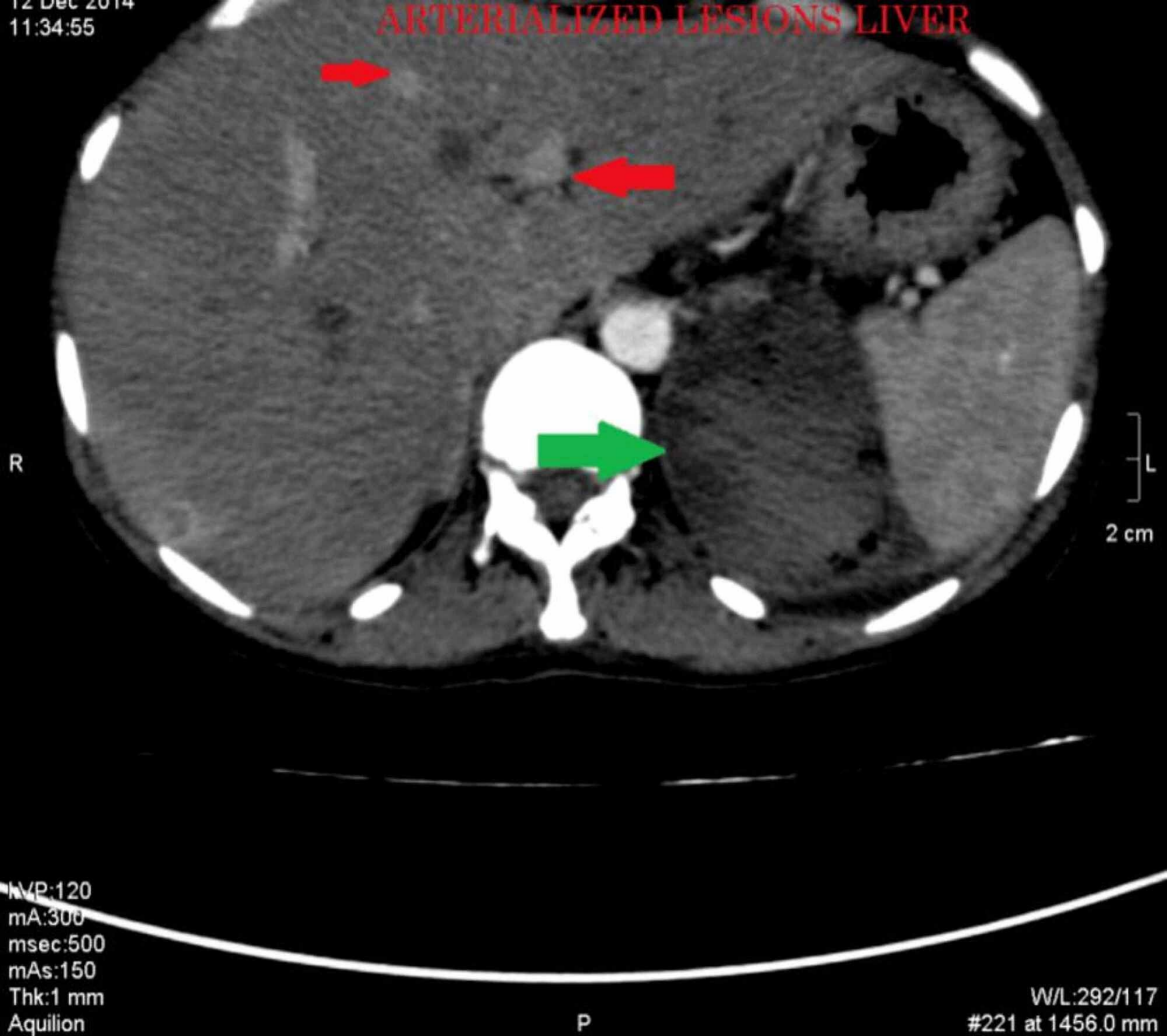
CECT abdomen axial section, arterial phase showing multiple arterialized lesions in the liver (red arrows) and a left subcapsular hematoma (green arrow) CECT: contrast-enhanced computed tomography

**Figure 8:**
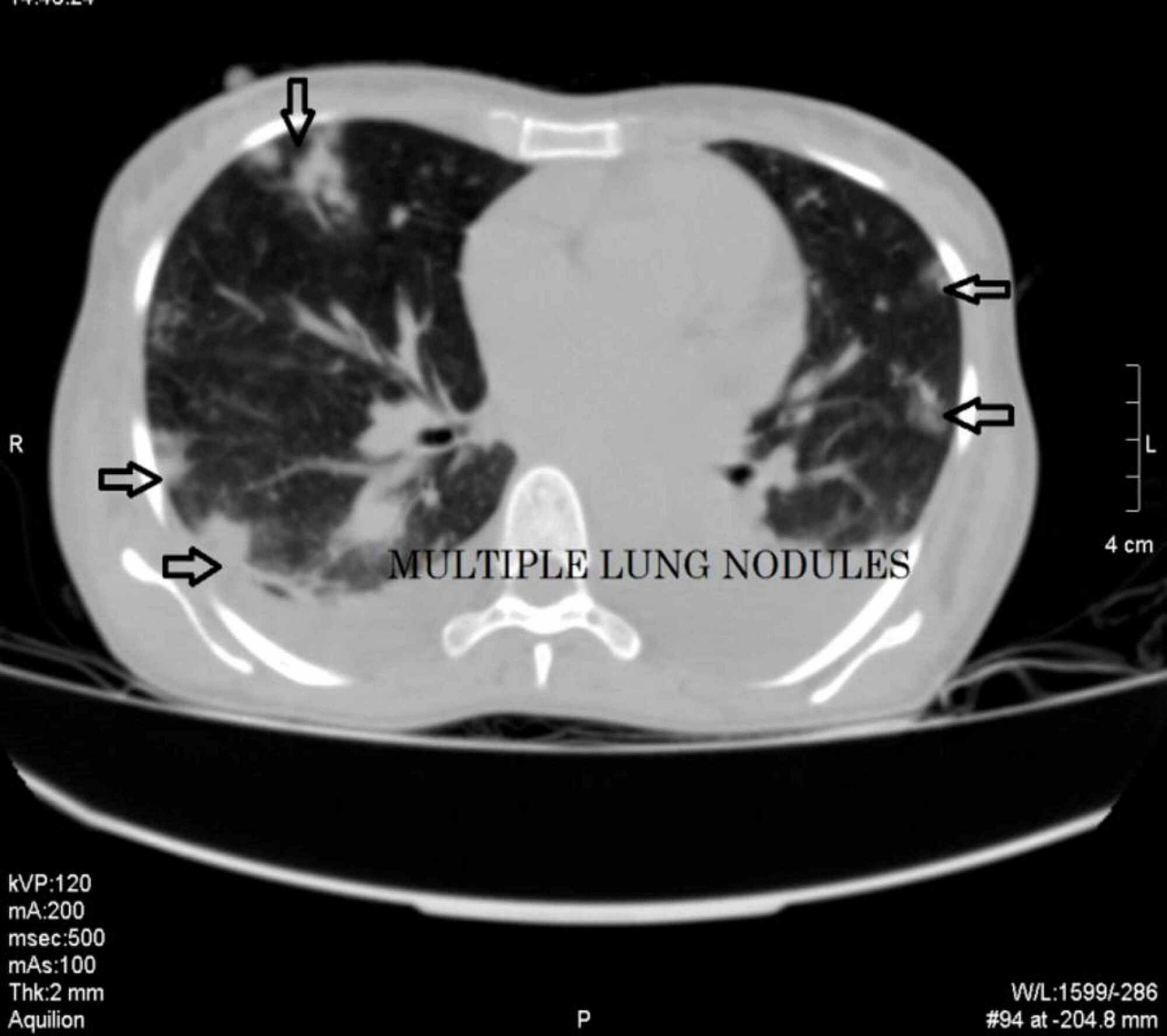
CT chest lung window showing multiple pulmonary nodules and bilateral pleural effusion CT: computed tomography

**Figure 9:**
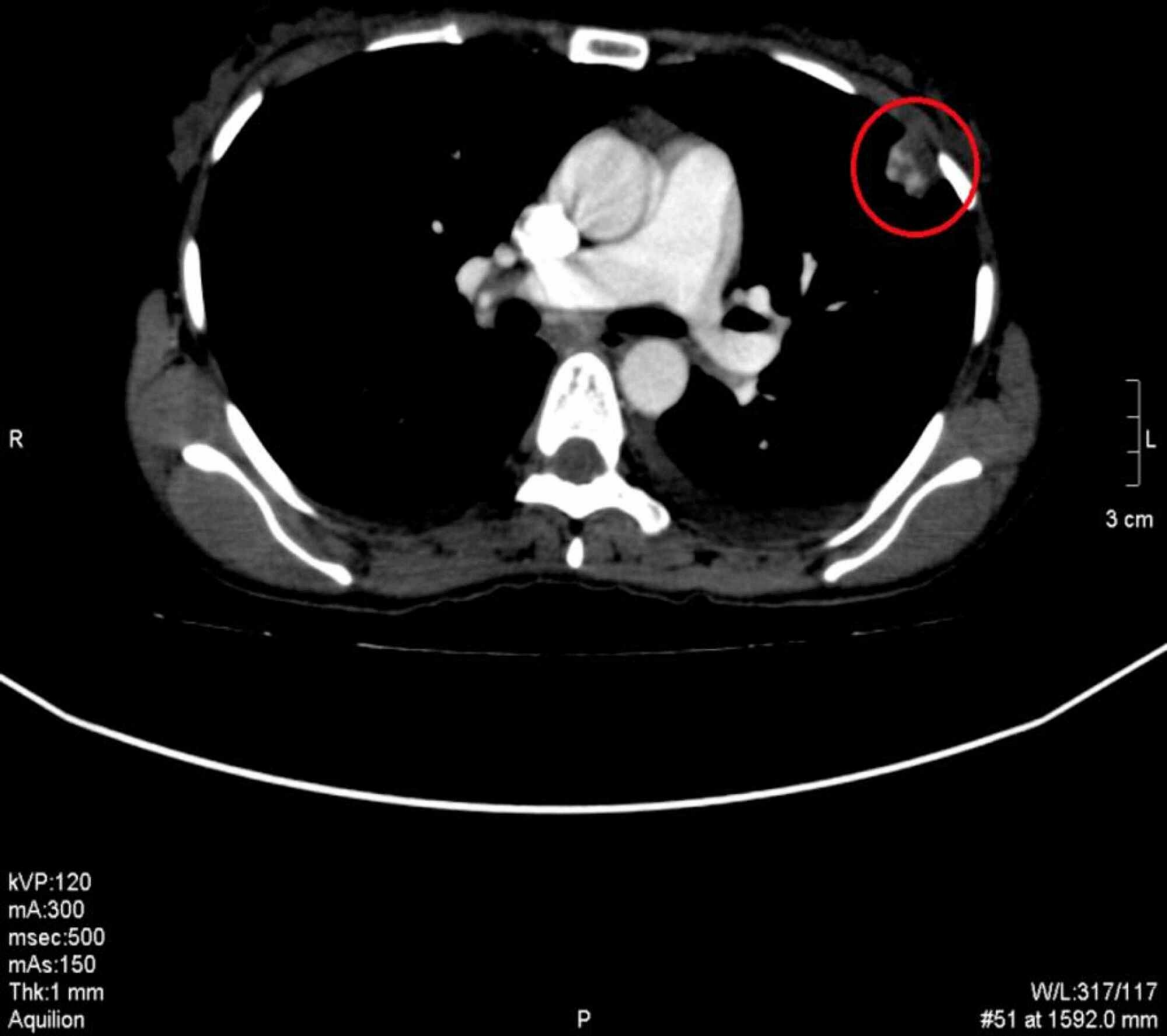
CECT chest, mediastinal window shows the vascular nature of the pulmonary nodule CECT: contrast-enhanced computed tomography

We did CT chest, abdomen, and pelvis, which showed an enhancing left ovarian mass measuring approximately 86 x 82 mm, associated with vascular lesions in both kidneys and the liver, multiple vascular pulmonary nodules, and bilateral pleural effusion (Figures 3-9).

**Figure 10:**
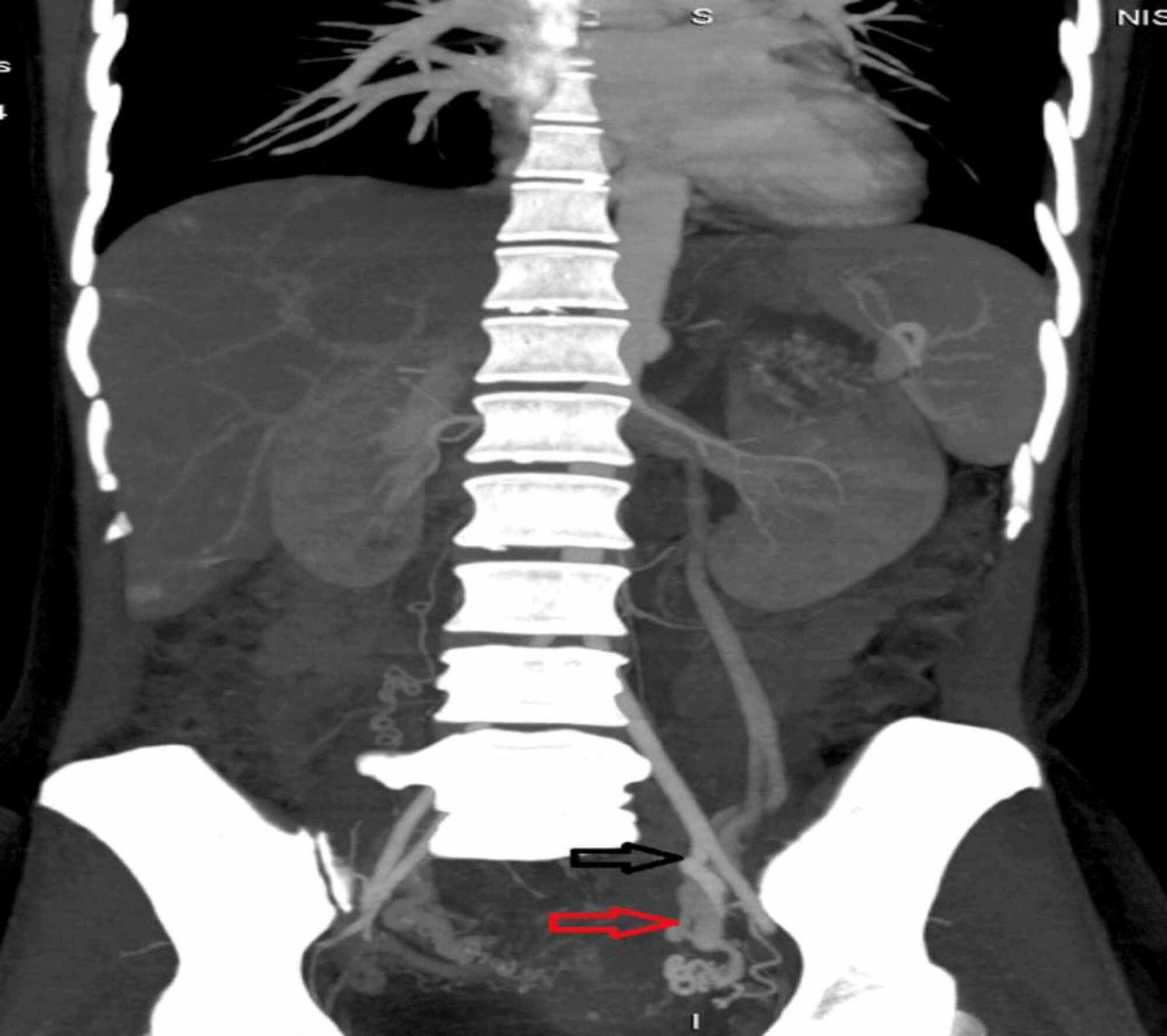
CT angiogram showing the mass to be hypervascular and fed by branches of the left internal iliac artery (black arrow) and drained by the left ovarian vein (red arrow) CT: computed tomography

CT angiogram showed the adnexal mass to be hypervascular, fed by branches of the left internal iliac artery, and drained by the left ovarian vein (Figure 10).

**Figure 11:**
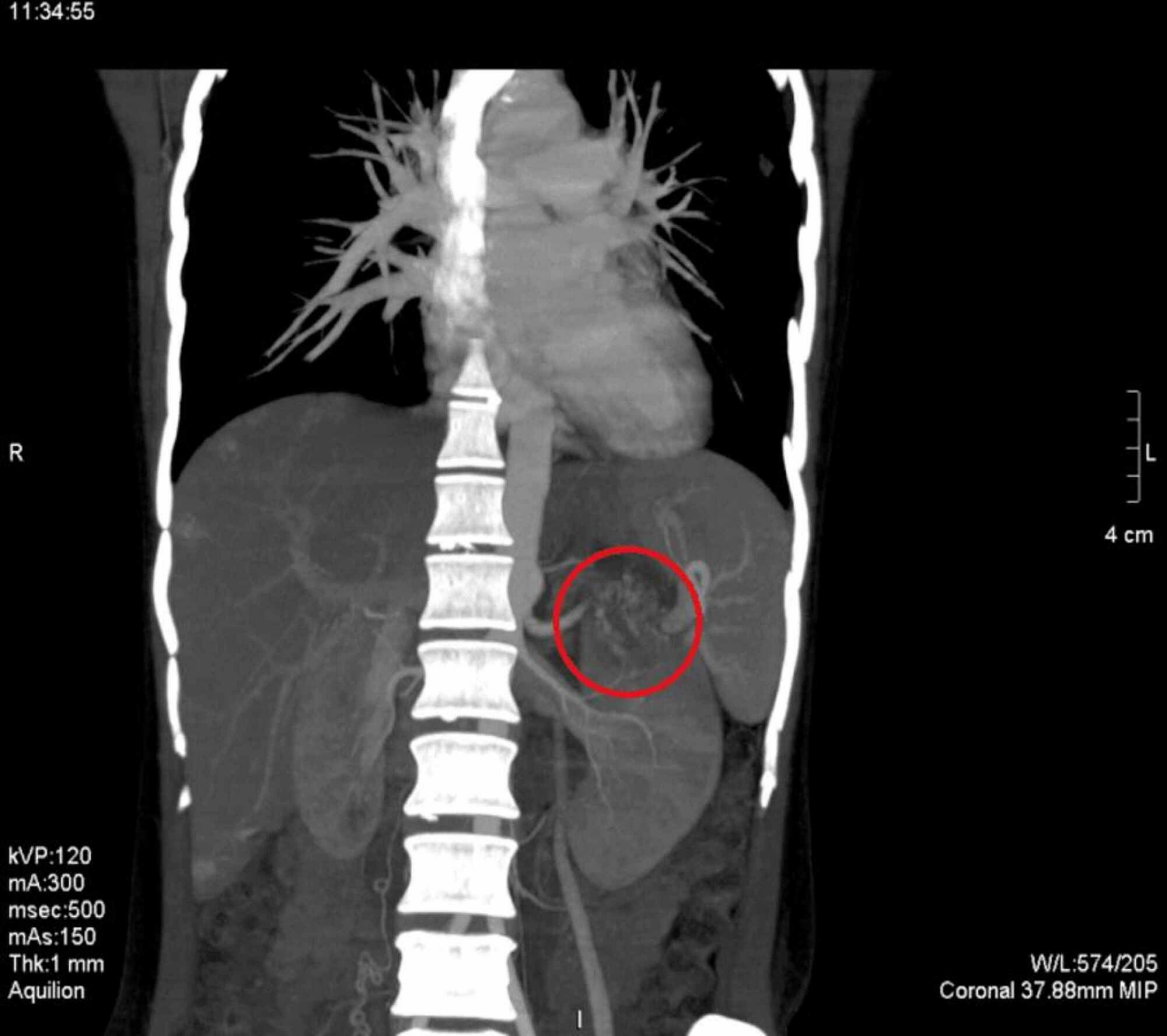
CT angiography shows the renal lesion to be hypervascular and fed by the branches from the aorta CT: computed tomography

The kidneys' lesions were supplied by branches from the abdominal aorta, as shown by CT angiography (Figure 11).

**Figure 12:**
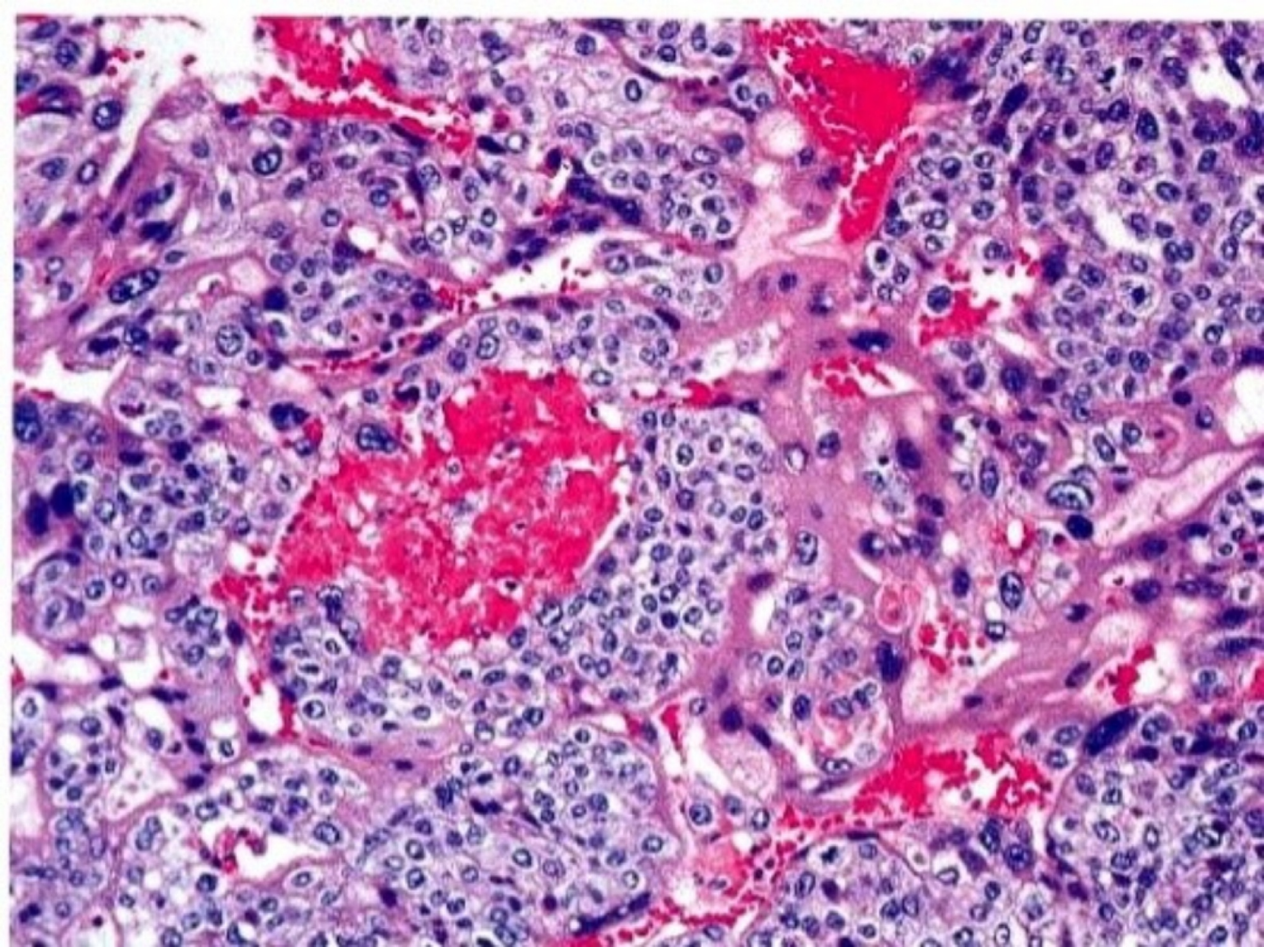
Microphotographs reveal cellular neoplasm with plump tumor cells in broad trabeculae separated by zones of hemorrhage and necrosis

The surgeon released the tumor from its adhesions. She performed a left ovarian cystectomy, right salpingo-oophorectomy, and omentectomy. Histopathological examination revealed cellular neoplasm with tumor cells separated by hemorrhage zones and necrosis (Figure 12). A cut section of the ovary showed a gray-white tumor with areas of hemorrhage and necrosis.

Her treatment regimen consisted of six cycles of chemotherapy with bleomycin, vincristine, and etoposide. She was on regular follow‑up with human chorionic gonadotropin (HCG) levels, which came down to 1800 IU/ml after three chemotherapy cycles. She was unable to complete the therapy and died due to cerebral metastasis.

## Discussion

Choriocarcinoma is the most aggressive neoplasm of gestational trophoblastic diseases. It is because of its rapid growth and metastatic potential. There are two types of pure ovarian choriocarcinomas, gestational and non-gestational. The gestational type of ovarian choriocarcinoma can originate in any ectopic ovarian pregnancy or it can result as a metastasis from any distant site of choriocarcinoma. The non-gestational type is a rare germ cell tumor with trophoblastic differentiation [1]. The estimated incidence of gestational ovarian choriocarcinoma is one in 369 million pregnancies [5]. Non-gestational choriocarcinoma accounts for 0.6% or less of all ovarian neoplasms [6]. Intrauterine choriocarcinoma is the most common sub-type. Few cases involving some other anatomical sites such as the ovary, fallopian tube, and abdominopelvic cavity have also been reported [7-10].

The diagnosis of primary extra-uterine choriocarcinoma is challenging. There is no specific clinical manifestation attributable to ovarian choriocarcinoma. However, its presentation can mimic other pathologies like ovarian torsion and ectopic pregnancy [7-10]. Choriocarcinoma produces an enormous amount of beta-human chorionic gonadotropin (β-HCG). The value ranges from three to 100 times more than a normal pregnancy. Serial measurements of the β-HCG level are of great diagnostic value. It also measures response to the treatment [11-12]. In a normal pregnancy, we can observe an intrauterine gestational sac on transvaginal sonography [12].

The potential sites where choriocarcinoma can metastasize involve uterine adnexa, pelvic cavity, lungs, brain, and liver. Rarely, it can also metastasize to the fetus via placental tissue [13]. The metastasis is commonly hemorrhagic because of trophoblastic cells' innate capacity to invade and erode vessel walls [14].

Chemotherapy is the management of choice for choriocarcinoma, even in metastatic disease. It has a curative rate of approximately 95% [11,13]. Currently, four to six courses of cisplatin, etoposide, and bleomycin (PEB) regimen as conventional therapy are the therapeutic standard for disseminated ovarian germ cell tumor (GCT) with excellent activity and acceptable toxicity [15]. We recommend the early evaluation of all the female patients for ovarian carcinoma presenting with bleeding per vaginum. Early diagnosis can aid in better management of the patients as well as prevention from metastatic disease.

## Conclusions

Non-gestational choriocarcinoma is a very rare ovarian neoplasm that is highly aggressive and usually present with vascular metastasis at the time of presentation. It poses diagnostic challenges because it mimics other more common conditions in women of childbearing age. Early diagnosis is crucial because the tumor is chemo-sensitive and has an excellent five-year survival rate despite early metastasis.
